# Supplementary material for: Nutritional Composition, Efficacy, and Processing of Vigna angularis (Adzuki Bean) for the Human Diet: An Overview
Source: Molecules. 2022 Sep 17;27(18):6079. doi: 10.3390/molecules27186079 (PMC9506481; doi:10.3390/molecules27186079)
Supplement: Supplementary file 1 [file molecules-27-06079-s001.zip › molecules-1877662-supplementary.pdf]

Table S1 Reviews on *Vigna* sp. from 2017 to 2022.

Objective of Review	Key Aspects Captured	References
Analysing and valorising *Vigna unguiculata*, *Vigna subterranea* and *Vigna vexillata* by considering the perspective of human activity and what effects it exerts.	Origin of species and time and place of domestication; the wild-type genome hides a valuable gene pool, and the process of species domestication will result in the loss of gene diversity.	Panzeri et al., 2022[1]
The opportunity for Bambara peanuts (*Vigna subterranea*) to address food insecurity and its benefits to local farmers in Africa.	Bambara peanuts's potential impact on global food production; Advances in next generation sequencing (NGS) technology improve peanut yields.	Olanrewaju et al., 2022[2]
The potential ability of mung bean (*Vigna radiata*) polyphenols to prevent Alzheimer's disease.	The effective dose and mechanism of mung bean polyphenols in the rodent model of Alzheimer's disease; Changes of neuroprotective phenolics in mung bean after germination.	Xu et al., 2021[3]
The nutritional composition, antinutritional components and health benefits of green gram (*Vigna radiata*).	Mung bean is rich in nutrients; The potential health benefits of mung beans; Mung bean has prebiotic and nutraceutical properties; Functions of mung bean in cosmetics and land reclamation.	Mekkara et al., 2021[4]
Current status of Yellow Mosaic Disease (YMD) in mung beans (*Vigna radiata*) and management opportunities.	The role of various begomoviruses, its genomic components, and vector whiteflies; Information about the genetics of YMD in both mungbean and blackgram crops is comprehensively presented; Implications of various management strategies; The prospects of employing various powerful emerging tools.	Mishra et al., 2020[5]
Introduction, breeding and germplasm evaluation of cowpea (*Vigna unguiculata*).	Useful characteristics of wild cowpeas; Interspecific crosses and backcross breeding in cowpea; Development of populations with introgressions from CWRs; Characterization and evaluation of populations with introgressions from CWRs.	Boukar et al.,2020[6]
The potential role of Bambara groundnut (*Vigna subterranean*) in diversifying agri-food systems and contributing to enhanced dietary and planetary sustainability.	Closing the food supply gap through improved production of Bambara groundnut; Closing the nutrient gap through enhanced utilization of Bambara groundnut.	Tan et al 2020[7]
Describe the beneficial potential of Bambara groundnut (*Vigna subterranean*) to protect and promote its cultivation as a food and medicine.	Background information and uses of Bambara groundnut; Description of the antimicrobial properties of Bambara groundnut.	Udeh et al., 2020[8]
Comprehensive evaluation of species information, nutritional composition and genetic breeding of rice beans (*Vigna umbellata*).	Sources of information and methodology of rice bean; Origin, distribution, taxonomy, morphology and composition of rice bean; Genetic, genomic resources and crop improvement of rice bean.	Pattanayak et al., 2019[9]

A comprehensive assessment of biotic stresses, abiotic stresses, and effective genetic improvement in mung bean (*Vigna radiata*).	The major diseases of mung bean; Key abiotic stresses affecting mung bean production; The challenges in genetic improvement.	Nair et al., 2019[10]
A comprehensive evaluation of mung bean (*Vigna radiata*) polyphenols, polysaccharides, peptides and health benefits.	Description of bioactive substances such as polyphenols, polysaccharides and peptides; health benefits of mung bean bioactive substances.	Hou et al., 2019[11]
Evaluate the potential for Bambara peanuts (*Vigna subterranean*) to be used to make a greater contribution to agriculture in preparation for climate change.	Evaluation of important traits of Bambara groundnut; Germplasm resources of Bambara groundnut for future breeding.	Mayes et al., 2019[12]
Description of the nutritional facts and health benefits of cowpeas (*Vigna unguiculata*).	Nutritional and functional compounds of cowpeas; Effect of processing on functional compounds in cowpea; Functional properties of cowpeas.	Jayathilake et al., 2018[13]
Discussions on different traits in mung bean (*Vigna radiata*) involved in defense against bruchids and their utility in pest management.	Bruchids infesting mung bean and their control; Sources of bruchid resistance in mung bean; Physical, biochemical and biochemical basis of resistance; Breeding constraints for developing bruchid-resistant mung bean.	War et al., 2017[14]
An overview of different aspects of cowpea, with a special emphasis on the molecular markers for assessing genetic diversity, as well as on biochemical and transcriptomic data with respect to evaluating cowpea drought stress tolerance.	Classification and description; Origin, domestication and distribution; evaluation of genetic diversity; Tolerance to drought stress on biochemical and transcriptomic data.	Carvalho et al., 2017[15]

References

1. Panzeri, D.; Nissim, W.G.; Labra, M.; Grassi, F. Revisiting the Domestication Process of African Vigna Species (Fabaceae): Background, Perspectives and Challenges. *Plants* **2022**, *11*, 532.
2. Olanrewaju, O.S.; Oyatomi, O.; Babalola, O.O.; Abberton, M. Breeding Potentials of Bambara Groundnut for Food and Nutrition Security in the Face of Climate Change. *Front.* Plant *Sci.* **2022**, *12*, 798993.
3. Xu, H.; Zhou, Q.; Liu, B.; Cheng, K.; Chen, F.; Wang, M. Neuroprotective Potential of Mung Bean (*Vigna radiata* L.) Polyphenols in Alzheimer's Disease: A Review. *J. Agric.* Food *Chem.* **2021**, *69*, 11554–11571.
4. Sudhakaran, S.M.N.; Bukkan, D.S. A review on nutritional composition, antinutritional components and health benefits of green gram (*Vigna radiata* (L.) Wilczek). *J. Food Biochem.* **2021**, *45*, e13743.
5. Mishra, G.P.; Dikshit, H.K.; Sv, R.; Tripathi, K.; Kumar, R.R.; Aski, M.; Singh, A.; Roy, A.; Kumari, N.; Dasgupta, U.; et al. Yellow Mosaic Disease (YMD) of Mungbean (*Vigna radiata* (L.) Wilczek): Current Status and Management Opportunities. *Front*. Plant *Sci.* **2020**, *11*, 918.
6. Boukar, O.; Abberton, M.; Oyatomi, O.; Togola, A.; Tripathi, L.; Fatokun, C. Introgression Breeding in Cowpea [*Vigna unguiculata* (L.) Walp.]. *Front*. Plant S*ci.* **2020**, *11*, 567425.
7. Tan, X.L.; Azam-Ali, S.; Goh, E.V.; Mustafa, M.; Chai, H.H.; Ho, W.K.; Mayes, S.; Mabhaudhi, T.; Azam-Ali, S.; Massawe, F. Bambara Groundnut: An Underutilized Leguminous Crop for Global Food Security and Nutrition. *Front. Nutr.* **2020**, *7*, 601496.
8. Udeh, E.L.; Nyila, M.A.; Kanu, S.A. Nutraceutical and antimicrobial potentials of Bambara groundnut (*Vigna subterranean*): A review. *Heliyon* **2020**, *6*, e5205.
9. Pattanayak, A.; Roy, S.; Sood, S.; Iangrai, B.; Banerjee, A.; Gupta, S.; Joshi, D.C. Rice bean: A lesser known pulse with well-recognized potential. *Planta* **2019**, *250*, 873–890.
10. Nair, R.M.; Pandey, A.K.; War, A.R.; Hanumantharao, B.; Shwe, T.; Alam, A.; Pratap, A.; Malik, S.R.; Karimi, R.; Mbeyagala, E.K.; et al. Biotic and Abiotic Constraints in Mungbean Production—Progress in Genetic Improvement. *Front*. Plant *Sci.* **2019**, *10*, 1340.
11. Hou, D.; Yousaf, L.; Xue, Y.; Hu, J.; Wu, J.; Hu, X.; Feng, N.; Shen, Q. Mung Bean (*Vigna radiata* L.): Bioactive Polyphenols, Polysaccharides, Peptides, and Health Benefits. *Nutrients* **2019**, *11*, 1238.

12. Mayes, S.; Ho, W.K.; Chai, H.H.; Gao, X.; Kundy, A.C.; Mateva, K.I.; Zahrulakmal, M.; Hahiree, M.K.I.M.; Kendabie, P.; Licea, L.C.S.; et al. Bambara groundnut: An exemplar underutilised legume for resilience under climate change. *Planta* **2019**, *250*, 803–820.
13. Jayathilake, C.; Visvanathan, R.; Deen, A.; Bangamuwage, R.; Jayawardana, B.C.; Nammi, S.; Liyanage, R. Cowpea: An overview on its nutritional facts and health benefits. *J. Sci. Food Agric.* **2018**, *98*, 4793–4806.
14. War, A.R.; Murugesan, S.; Boddepalli, V.N.; Srinivasan, R.; Nair, R.M. Mechanism of Resistance in Mungbean [*Vigna radiata* (L.) R. Wilczek var. radiata] to bruchids, *Callosobruchus* spp. (*Coleoptera: Bruchidae*). *Front.* Plant *Sci.* **2017**, *8*, 1031.
15. Carvalho, M.; Lino-Neto, T.; Rosa, E.; Carnide, V. Cowpea: A legume crop for a challenging environment. *J. Sci. Food Agric.* **2017**, *97*, 4273–4284.
